# A Concise Review on the Role of CircPVT1 in Tumorigenesis, Drug Sensitivity, and Cancer Prognosis

**DOI:** 10.3389/fonc.2021.762960

**Published:** 2021-11-04

**Authors:** Soudeh Ghafouri-Fard, Tayyebeh Khoshbakht, Mohammad Taheri, Elena Jamali

**Affiliations:** ^1^ Department of Medical Genetics, School of Medicine, Shahid Beheshti University of Medical Sciences, Tehran, Iran; ^2^ Phytochemistry Research Center, Shahid Beheshti University of Medical Sciences, Tehran, Iran; ^3^ Urology and Nephrology Research Center, Shahid Beheshti University of Medical Sciences, Tehran, Iran; ^4^ Institute of Human Genetics, Jena University Hospital, Jena, Germany; ^5^ Skull Base Research Center, Loghman Hakim Hospital, Shahid Beheshti University of Medical Sciences, Tehran, Iran; ^6^ Department of Pathology, Loghman Hakim Hospital, Shahid Beheshti University of Medical Sciences, Tehran, Iran

**Keywords:** circRNA, circPVT1, cancer, expression, biomarker

## Abstract

CircPVT1 (hsa_circ_0001821) is a cancer-related circular RNA (circRNA) that originated from a genomic locus on chromosome 8q24. This locus has been previously found to encode the oncogenic long non-coding RNA PVT1. Expression of this circRNA has been found to be upregulated in diverse neoplastic conditions. CircPVT1 acts as a sponge for miR-125a, miR-125b, miR-124-3p, miR-30a-5p, miR-205-5p, miR‐423‐5p, miR‐526b, miR-137, miR-145-5p, miR-497, miR-30d/e, miR-455-5p, miR-29a-3p, miR-204-5p, miR-149, miR-106a-5p, miR-377, miR-3666, miR-203, and miR-199a-5p. Moreover, it can regulate the activities of PI3K/AKT, Wnt5a/Ror2, E2F2, and HIF-1α. Upregulation of circPVT1 has been correlated with decreased survival of patients with different cancer types. In the current review, we explain the oncogenic impact of circPVT1 in different tissues based on evidence from *in vitro*, *in vivo*, and clinical investigations.

## Introduction

Circular RNAs (circRNAs) are single-stranded covalently enclosed uninterrupted loops with no free end or polyadenylated tail (1). These transcripts are prevalent in human transcriptome since approximately 20% of active genes have the potential to produce circRNAs (1, 2). In fact, circRNAs are a group of long non-coding RNAs (lncRNAs). Compared with linear ncRNA, circRNAs have more stability, since their circular structure protects them from degradation by the majority of RNA decay mechanisms (3, 4).

Being mainly produced by back-splicing, circRNAs consist of exonic and/or intronic regions. Back-splicing is a non-canonical alternative RNA splicing process that is facilitated by the spliceosomes and contribution of a number of *cis*- or *trans*-acting factors (5). Biogenesis of circRNA is under control of numerous *cis*- and *trans*-acting factors (6). The splice sites, enhancers, and silencers, particularly element adjacent to the junction sites, including the inverted Alu repeat segments are examples of the *cis*-regulatory factors (7). Spliceosome elements, RNA helicases, and RNA-binding proteins are among *trans*-regulatory factors in regulation of circRNA biogenesis (8).

CircRNAs have functional roles in the regulation of gene expression through competitively binding and sponging miRNAs. This action of circRNAs leads to the stabilization of miRNA targets (5). This mode of action of circRNAs is well assessed. In fact, a number of circRNAs have numerous binding sites for single or numerous miRNAs (5). In addition, a number of circRNAs can sponge proteins and block their activity (9). Some circRNAs can bind to numerous proteins and keep them together. These circRNAs serve as a scaffold to enable interactions of these proteins (5). Thus, in addition to sponging miRNAs, they regulate gene expression *via* interacting with several proteins. There is also evidence that certain circRNAs can produce proteins (10).

Three major categories of circRNA have been identified: exonic circRNAs, circular intronic RNAs, and exon–intron circRNAs (11). Exonic circRNAs mostly serve as miRNA sponges. Thus, they increase expression of miRNA targets through adsorption of miRNAs. However, intron-containing circRNAs (including both circular intronic RNAs and exon–intron circRNAs) are mainly located in the nucleus where they modulate transcription of certain genes (12, 13).

CircRNAs partake in the regulation of all principal hallmarks of malignancy and are considered as promising markers for diagnosis and prediction of course of cancer (5).

CircPVT1 (hsa_circ_0001821) is an example of a cancer-related circRNA that originated from a genomic locus on chromosome 8q24 (14). This locus has been previously found to encode the oncogenic lncRNA PVT1. The CircInteractome Database (https://circinteractome.nia.nih.gov/index.html) has listed 26 isoforms for circPVT1 (15). The spliced length of these isoforms ranges from 113 to more than 11,000 nucleotides, with the most frequent isoform being 410 nucleotide long. CircPVT1 is produced by back-splicing and encompasses the entire length of exon 2 of PVT1 (16). On the other hand, some of the identified 26 isoforms of lncRNA PVT1 do not have exon 2 (17). These alternatively spliced variants have 5′ cap and polyadenylated tail at 3′ end (18).

Expression of circPVT1 has been assessed by different methods. Current methods usually use simple statistical methods or differential expression analysis strategies developed for linear RNAs. As the majority of circRNAs have very low levels of expression, RNase R treatment is typically used for enrichment of circRNAs. When RiboMinus/RNase R-treated RNA-seq libraries are used, alterations in enrichment coefficient in the RNase R treatment phase might lead to bias in estimation of circRNA expression (19).

A high-throughput RNA sequencing experiment for comparison of circRNA signature in proliferating versus senescent human fibroblasts has identified circPVT1 as a downregulated transcript in senescent fibroblasts. Further experiments have indicated that downregulation of circPVT1 expression in proliferating fibroblasts induces their senescence, as being evident by upregulation of senescence-related β-galactosidase level, upregulation of CDKN1A/P21 and TP53, and decrease in proliferation rate. These effects are most probably mediated through modulation of let-7 levels and consequent alteration in levels of let-7-regulated transcripts, including IGF2BP1, KRAS, and HMGA2 (16).

Among different cancer types, circPVT1 has been primarily identified as an upregulated circRNA in gastric cancer specimens compared with corresponding normal tissues (14). Subsequently, overexpression of this circRNA has been verified in other types of malignancies. In this review, we explain the oncogenic roles of circPVT1 in different tissues based on evidence from *in vitro*, *in vivo*, and clinical investigation.

## *In Vitro* Studies

CircPVT1 has been found to increase proliferation of gastric cancer cell through serving as a molecular sponge for miR-125 family members (14). Moreover, expression of circPVT1 has been higher in paclitaxel-resistant gastric cancer cells. CircPVT1 silencing has improved sensitivity of gastric cancer cells through modulating miR-124-3p levels. Since ZEB1 is a direct target of miR-124-3p, circPVT1 enhances expression of ZEB1 through sequestering this miRNA (20). Exosomal levels of circPVT1 have also been higher in cisplatin-resistant gastric cancer cells parallel with downregulation of miR-30a-5p. CircPVT1 silencing has suppressed cisplatin resistance of gastric cancer cells through inducing apoptosis and reducing invasion or autophagy. Functionally, circPVT1 modulates expression of YAP1 through influencing expression of miR-30a-5p (21).

In breast cancer cells, upregulation of circPVT1 has been accompanied with underexpression of miR-29a-3p. Suppression of circPVT1 or upregulation of miR-29a-3p could block proliferation, invasiveness, and migratory potential of breast cancer cells while promoting their apoptosis. Mechanistically, circPVT1 binds with miR-29a-3p to release AGR2 from its inhibitory effect. AGR2 has been found to increase expression of HIF-1α and then accelerated malignant features of breast cancer cells (22). CircPVT1 has also been shown to promote invasiveness and epithelial–mesenchymal transition (EMT) of neoplastic breast cells through sequestering miR-204-5p (23).

Expression of circPVT1 has also been upregulated in human epithelial ovarian cancer cells. In both SKOV3 and CAOV3 cells, suppression of circPVT1 has decreased cell proliferation and enhanced cell apoptosis. CircPVT1 has been found to negatively regulate miR-149 (24).

CircPVT1 has been demonstrated to be upregulated in osteosarcoma cells parallel with upregulation of c-FLIP and downregulation of miR-205-5p. CircPVT1 silencing has suppressed proliferation, migration, and invasiveness of osteosarcoma cells through inhibiting EMT. This circRNA sponges miR-205-5p and increases expression of c-FLIP (25). Another study in osteosarcoma has revealed downregulation of miR-423-5p while upregulation of Wnt5a/Ror2 and circPVT1. MiR-423-5p has a role in inhibition of glycolysis and suppression of cell proliferation, migration, and invasiveness through influencing expressions of Wnt5a and Ror2. CircPVT1-mediated silencing of miR-423-5p leads to activation of Wnt5a/Ror2 signaling (26). CircPVT1 also enhances metastasis of osteosarcoma through modulation of miR‐526b/FOXC2 axis (27).

In addition, circPVT1 affects response of osteosarcoma cells to chemotherapeutic medications since its silencing has decreased chemoresistance of osteosarcoma cells to doxorubicin and cisplatin through reducing levels of ABCB1 (28). CircPVT1 also participates in doxorubicin resistance of these cells through miR-137–TRIAP1 axis (29).

CircPVT1 contributes in the malignant behaviors of lung cancer *via* different routes. It induces chemoresistance *via* modulation of the miR-145-5p/ABCC1 signals (30). In addition, it enhances proliferation and invasion of lung cancer cells *via* sequestering miR-125b and enhancing E2F2 signals (31). CircPVT1 also serves as a sponge for miR-497 to increase levels of Bcl-2 lung cancer cells (32).

In oral squamous cell carcinoma, circPVT1 has been found to sponge miR-125b and miR-106a-5p to release STAT3 and HK2 from their inhibitory effects (33, 34). In acute lymphoblastic leukemia, the oncogenic role of circPVT1 is mediated through upregulation of Bcl-2 and c-Myc (35).

In hepatocellular carcinoma, circPVT1 regulates proliferation as well as apoptotic and glycolytic processes through modulation of miR-377/TRIM23 axis (36). Moreover, it regulates cell growth *via* modulation of expression of miR-3666 and Sirtuin 7 (37). MiR-203/HOXD3 is another molecular axis being regulated by circPVT1 in hepatocellular carcinoma (38).

Finally, miR-199a-5p and miR‐145‐5p have been identified as targets of circPVT1 in glioblastoma (39) and clear cell renal cell carcinoma (40), respectively.

**Figure 1:**
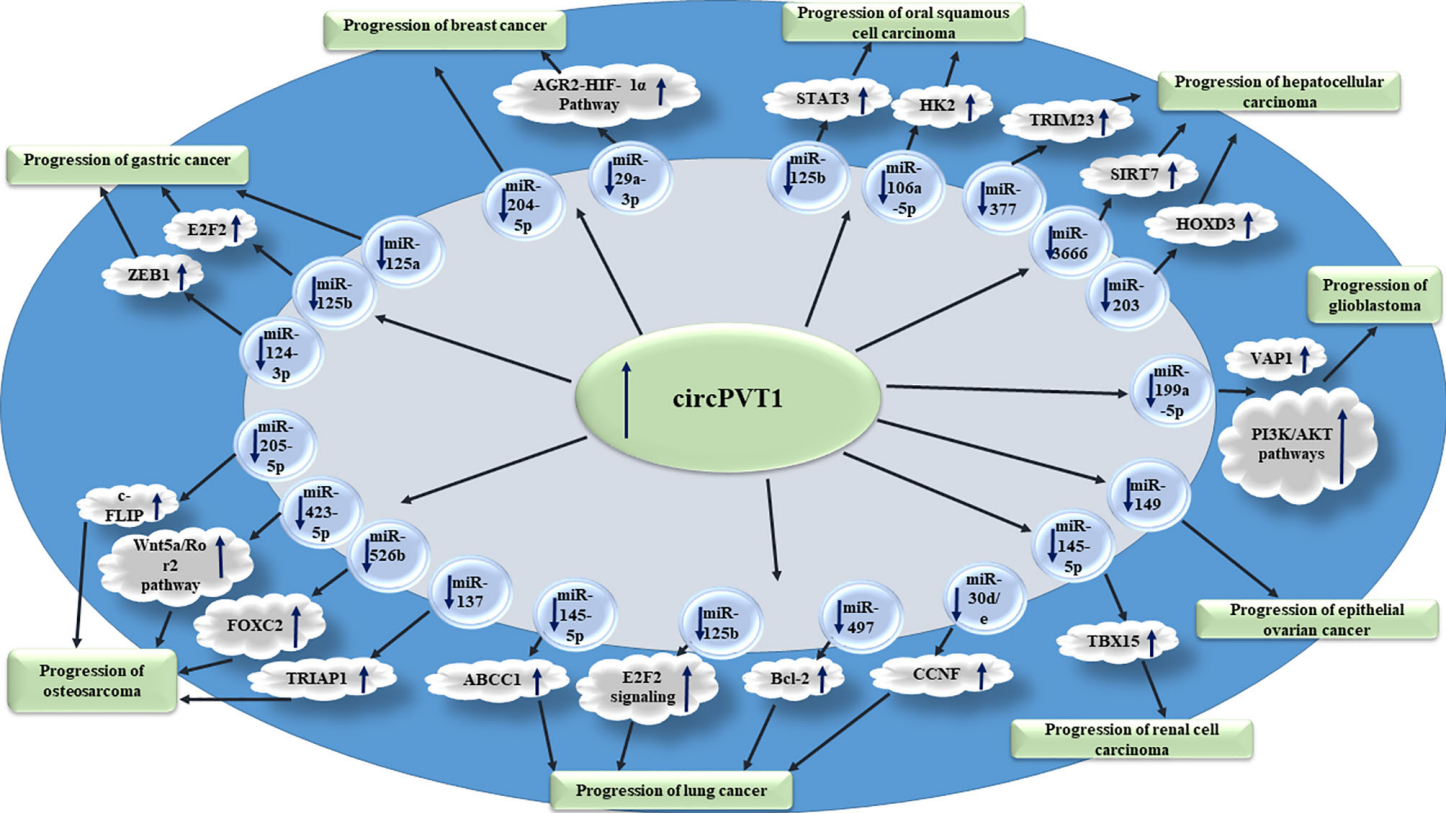
Oncogenic roles of circPVT1 in different cancer types are mainly exerted through sponging miRNAs.

**Figure 1** shows the oncogenic roles of circPVT1 in different cancer types.

**Table 1** shows the impact of circPVT1 in carcinogenesis based on *in vitro* studies.

**Table 1:** Impact of circPVT1 carcinogenesis based on cell line studies.

Tumor type	Targets/regulators and signaling pathways	Cell line	Function	Reference
Acute lymphoblastic leukemia	c-Myc, Bcl-2	GES-1, Nalm-6 B-ALL, LO2, BEL-7402, Hep3B, HepG2	∆ circPVT1: ↓ proliferation, ↑ apoptosis	(35)
Breast cancer	MiR-29a-3p, AGR2-HIF-1α Pathway	MDA-MB-231, MCF7	∆ circPVT1: ↓ proliferation, ↓ migration, ↓ invasion, ↑ apoptosis ↑ circPVT1: ↑ proliferation, ↑ migration, ↑ invasion ↓ apoptosis	(22)
	MiR-204-5p	MDA-MB-231, MDA-MB-468, MCF-7, MCF-10A	∆ circPVT1: ↓ proliferation, ↓ migration, ↓ invasion, ↓ EMT process, ↑ apoptosis	(23)
Cutaneous squamous cell carcinoma	Not reported	HaCat, A431, SCL-1, and SCL-12	∆ circPVT1: ↓ migration, ↓ invasion	(41)
Epithelial ovarian cancer	MiR-149	CAOV3, SKOV3, OVCAR3, SNU119	∆ circPVT1: ↓ proliferation, ↑ apoptosis	(24)
Esophageal cancer	MiR-4663, Pax-4, Pax-6, PPARα, PPAR-γ	EC109, CaES-17, TE-1, TE-10, HEEC, HepG2, MKN45, SW60, A549	∆ circPVT1: ↓ proliferation ↑ circPVT1: ↑ invasion	(42)
Gastric cancer (GC)	MiR-125a, miR-125b, E2F2	MGC-803, AGS, HEK-293T	∆ circPVT1: ↓ proliferation	(14)
	MiR-124-3p, ZEB1	MKN-45, HGC-27, MGC-803, and AGS, GES-1	∆ circPVT1: ↑ PTX sensitivity	(20)
	MiR-30a-5p, YAP1	GES-1, HGC-27, AGS	∆ circPVT1: ↓ DDP resistance, ↓ invasion, ↓ autophagy, ↑ apoptosis	(21)
Glioblastoma	MiR-199a-5p, VAP1, PI3K/AKT pathways	U539, U251	∆ circPVT1: ↓ proliferation, ↓ migration, ↓ EMT process, ↑ apoptosis	(39)
Hepatocellular carcinoma	MiR-377, TRIM23	THLE-2, SNU-387, Huh7	∆ circPVT1: ↓ proliferation, ↓ glycolysis, ↑ apoptosis	(36)
	MiR-3666, SIRT7	HL-7702, SKHEP-1, SMMC-7721, HepG2, MHCC97H, 293T	∆ circPVT1: ↓ proliferation, ↓ colony formation, ↑ apoptosis	(37)
	MiR-203, HOXD3	Huh7, Sk-hep1, SMMC-7721, HepG2, L-02	∆ circPVT1: ↓ proliferation, ↓ migration	(38)
Lung cancer	MiR-145-5p, ABCC1	PAEC, PC9, A549	∆ circPVT1: ↑ cisplatin and pemetrexed sensitivity	(30)
	MiR-125b, E2F2 signaling pathway, c-Fos	A549, H292, SPC-A1, H1299, H1650, H1975, SK-MES-1, HBE	∆ circPVT1: ↓ proliferation, ↓ invasion	(31)
	MiR-497, Bcl-2	H1299, H1650, A549, PC9, SK-MES-1, 16HBE	∆ circPVT1: ↓ proliferation, ↑ apoptosis	(43)
	MiR-30d/e, CCNF	A549, H520, H226, SKMES-1, H1270	∆ circPVT1: ↓ proliferation,	(44)
Medullary thyroid cancer	MiR-455-5p, CXCL12	TT, MZ-CRC-1, NThyy-ori 3.1	∆ circPVT1: ↓ proliferation, ↓ migration, ↓ invasion	(45)
Oral squamous cell carcinoma	MiR-125b, STAT3	SCC-9, CAL-27, HOK	∆ circPVT1: ↓ proliferation	(43)
	MiR-106a-5p, HK2	HNOK, SCC15, SCC9, CAL-27, SCC4	∆ circPVT1: ↓ viability, ↓ migration, ↓ invasion, ↓ glycolytic metabolism, ↑ apoptosis	(34)
Osteosarcoma (OS)	ABCB1	Saos-2, KHOS, U2OS, MG63	∆ circPVT1: ↓ doxorubicin and cisplatin resistance	(28)
	MiR-205-5p, c-FLIP	Saos-2, MG63, U2OS, SW1353, hFOB 1.19	∆ circPVT1: ↓ proliferation, ↓ migration, ↓ invasion, ↓ EMT process	(25)
	MiR‐423‐5p, Wnt5a/Ror2 pathway	MG‐63, Saos‐2, HOS, and U2OS, hFOB 1.19	∆ circPVT1: ↓ proliferation, ↓ migration, ↓ invasion, ↓ glycolysis	(46)
	MiR‐526b, FOXC2	hFOB 1.19, MG‐63, U2OS, HOS, 143B	∆ circPVT1: ↓ migration, ↓ invasion	(47)
	MiR-137, TRIAP1	hFOB 1.19, KHOS, U2OS, 293T	∆ circPVT1: ↑ DXR Sensitivity	(29)
Renal cell carcinoma	MiR‐145‐5p, TBX15	ACHN, 786‐O, Caki‐1, HK‐2, 293T	∆ circPVT1: ↓ proliferation, ↓ migration, ↓ invasion, ↑ G1 phase arrest, no significant difference in apoptosis	(40)

∆, knock-down or deletion; PTX, paclitaxel; DXR, doxorubicin; DDP, cisplatin.

## Animal Studies

Animal studies have shown the role of circPVT1 suppression on enhancement of cisplatin sensitivity of gastric cancer through miR-30a-5p/YAP1 axis (21). Moreover, circPVT1 silencing could increase drug sensitivity in osteosarcoma models (29). Other studies have consistently pointed to the fact that circPVT1 silencing decreases the ability of malignant cells in induction of palpable tumors in animal models. Almost all of these studies have used BALB/c mice as the recipient of cancer cells (**Table 2**).

**Table 2:** Impact of circPVT1 carcinogenesis based on animal studies.

Tumor type	Animal models	Results	Reference
Breast cancer	Male BALB/c nude mice	↑ circPVT1: ↑ tumor growth	(22)
	Male athymic BALB/c nude mice	∆ circPVT1: ↓ tumor weight, ↓ tumor growth	(23)
Gastric cancer	Male BALB/c nude mice	∆ circPVT1: ↓ tumor size, ↓ tumor weight, ↑ PTX sensitivity	(20)
	BALB/c nude mice	∆ circPVT1: ↓ tumor volume, ↓ tumor weight	(21)
Hepatocellular carcinoma	BALB/c nude mice	∆ circPVT1: ↓ tumor volume, ↓ tumor weight	(36)
	Male athymic BALB/c mice	↑ circPVT1: ↑ tumor volume, ↑ tumor growth	(38)
Lung cancer	Male BALB/c nude mice	↑ circPVT1: ↑ tumor growth	(31)
	Male athymic BALB/c nude mice	∆ circPVT1: ↓ tumor weight, ↓ tumor growth	(43)
	Nude mice	∆ circPVT1: ↓ tumor volume	(44)
Medullary thyroid cancer	Nude mice	∆ circPVT1: ↓ tumor volume, ↓ tumor growth	(45)
Oral squamous cell carcinoma	BALB/c nude mice	∆ circPVT1: ↓ tumor volume, ↓ tumor weight	(34)
Osteosarcoma	Nude mice	∆ circPVT1: ↓ tumor volume, ↓ tumor weight, ↓ metastasis	(46)
	Male BALB/c nude mice	∆ circPVT1: ↓ tumor volume, ↓ tumor weight, ↑ drug sensitivity	(29)
Renal cancer	Male BALB/c nude mice	↑ circPVT1: ↑ tumor volume, ↑ tumor weight, ↑ metastasis	(40)

∆, knock-down or deletion; PTX, paclitaxel.

## Human Studies

CircPVT1 levels have been found to be upregulated in gastric cancer tissues as a result of amplification of its genomic locus. Expression of circPVT1 could be regarded as an independent prognostic marker for prediction of overall and disease-free survival of patients with this type of cancer (14). Serum exosomal levels of circPVT1 have been higher in cisplatin-resistant gastric cancer patients, indicating a role for this circRNA in predicting response to cisplatin (21). CircPVT1 has also been found to be upregulated in both osteosarcoma tissues and serum samples of these patients in correlation with poor prognosis of osteosarcoma patients. Moreover, circPVT1 performance as a diagnostic marker for osteosarcoma has been superior to alkaline phosphatase (28). Another study in osteosarcoma patients has shown upregulation of circPVT1 in osteosarcoma tissues compared with normal tissues. Moreover, expression of circPVT1 has been considerably elevated in the chemoresistant patients compared with the chemosensitive ones (29). While the association between overexpression of circPVT1 and lymph node metastasis has been verified in gastric cancer (14) and colorectal cancer (48), Kong et al. reported no correlation between expression levels of circPVT1 and lymph node metastasis in gastric cancer (49). Moreover, they reported downregulation of circPVT1 in gastric cancer (49). Other studies in diverse types of cancers have verified overexpression of circPVT1 in neoplastic tissues versus non-neoplastic tissues adjacent to the tumors (**Table 3**). Upregulation of circPVT1 has been correlated with tumor size in non-small cell lung cancer (32) and hepatocellular carcinoma (38). However, this correlation has not been verified in osteosarcoma (27).

**Table 3:** Impact of circPVT1 carcinogenesis based on human studies.

Tumor type	Samples	Expression (tumor *vs.* normal)	Kaplan–Meier analysis (impact of circPVT1 upregulation)	Prognostic factors based onunivariate/multivariate Cox regression analyses	Association of circPVT1 expression with clinicopathologic characteristics	Reference
Acute lymphoblastic leukemia (ALL)	20 BM samples from AML patients, and 48 BM samples from ALL patients, and 40 controls	Higher in ALL but not AML	NR	NR	Age (in ALL)	(35)
Breast cancer (BC)	40 BC tissues and ANCTs	High	Poor survival and low median survival time	NR	Lymph node positivity and tumor size	(22)
	99 BC tissues and ANCTs	High	Worse OS	NR	advanced TNM stage	(23)
Cutaneous squamous cell carcinoma (CSCC)	30 pairs of CSCC tumor tissues and ANCTs	High	NR	NR	NR	(41)
Esophageal cancer	20 esophageal cancer patients and 20 healthy volunteers	No significant difference	NR	NR	NR	(42)
	20 tumor tissues and ANCTs	High	NR	NR	NR	
Gastric cancer (GC)	20 pairs of GC tissues and normal tissues	High	NR	NR	NR	(14)
	187 pairs of GC tissues and ANCTs	High	Longer OS and DFS	CircPVT1 expression, tumor size, and TNM stage (for OS and DFS)	Low circPVT1 expression associated with late T stage and positive neural invasion	
	30 PTX-sensitive patients and 30 PTX-resistant patients	Higher in PTX-resistant GC tissues than PTX-sensitive tissues	NR	NR	NR	(20)
	30 GC tissues	High	NR	NR	Tumor–node–metastasis grade, lymph node metastasis, tumor size, DDP resistance	(21)
Glioblastoma	25 GBM tissues and ANCTs	High	NR	NR	NR	(39)
Hepatocellular carcinoma (HCC)	26 pairs of HCC tissues and ANCTs	High	NR	NR	NR	(36)
	45 pairs of HCC tissues and ANCTs	High	NR	NR	NR	(37)
	70 pairs of HCC tissues and ANCTs	High	NR	NR	Overall survival, lymph node metastasis, and TNM stages	(38)
Lung cancer	104 LAD tissues and corresponding ANCTs	High	Shorter OS	CircPVT1 expression	N stage and chemotherapy insensitivity	(30)
	96 NSCLC patients and 96 healthy controls	High	Lower OS and chemotherapy-resistant	CircPVT1 expression and TNM stage	Negatively correlated with differentiation or p-TNM stage	(50)
	68 pairs of NSCLC tissues and ANCTs	High	NR	NR	Distant metastasis	(31)
	Serum of 45 NSCLC patients and 45 healthy controls	High	NR	NR	NR	
	90 pairs of NSCLC tissues and ANCTs	High	Shorter OS	NR	Tumor size and TNM stage	(43)
	8 LUSC tissues and 9 healthy lung samples	High	NR	NR	NR	(44)
	104 pairs of LUSC tissues and 110 pairs of serum samples	High	Worse OS	CircPVT1 expression (OS)	TNM stage, lymph node metastasis, and tumor size	
Medullary thyroid cancer (MTC)	28 MTC tissues and ANCTs	High	Lower OS	NR	NR	(45)
Oral squamous cell carcinoma (OSCC)	50 OSCC tissues and ANCTs	High	NR	NR	Tumor size and tumor, node, and metastasis	(43)
	30 pairs of OSCC tissues and ANCTs	High	NR	NR	_	(34)
Osteosarcoma	80 pairs of malignant tissues and ANCTs	High	Shorter OS	NR	Advanced Enneking stage, metastasis, and chemoresistance	(28)
	25 pairs of malignant tissues and corresponding ANCTs	High	NR	NR	NR	(25)
	36 pairs of malignant tissues and ANCTs	High	Lower OS	NR	NR	(46)
	48 pairs of malignant tissues and ANCTs	High	Shorter OS	NR	Advanced clinical stage, distant metastasis	(47)
	52 tumor patients and 45 normal samples	High	NR	NR	Chemoresistance	(29)
Renal cell carcinoma (RCC)	7 ccRCC tissues and ANCTs (GSE108735)	High	NR	NR	NR	(40)
	90 ccRCC tissues and ANCTs	High	NR	NR	T stage, N stage, and M stage	

PTX, paclitaxel; DDP, cisplatin; ANCTs, adjacent non-cancerous tissues; OS, overall survival; MVI, microvascular invasion; DFS, disease-free survival; TNM, tumor–node–metastasis; LAD, lung adenocarcinoma; NSCLC, non-small cell lung cancer; BM, bone marrow; AML, acute myelogenous leukemia; NR, not reported; GBM, glioblastoma; LUSC, lung squamous cell carcinoma; ccRCC, clear cell renal cell carcinoma.

In lung cancer, expression levels of circPVT1 could differentiate tumor samples from neighboring non-cancerous tissues with diagnostic power of 0.803. More importantly, serum levels of circPVT1 could diagnose patients from healthy subjects with diagnostic value of 0.794 (31). The diagnostic value of circPVT1 has also been assessed in oral squamous cell carcinoma tissue specimens through depicting receiver operating characteristic (ROC) curves. The area under this curve has been measured as 0.787 with sensitivity and specificity values of 68.6% and 86.0%, respectively (33) (**Table 4**).

**Table 4:** Diagnostic value of circPVT1 in cancers.

Tumor type	Samples	Distinguish between	Area under the curve	Sensitivity (%)	Specificity (%)	References
Lung cancer	68 pairs of NSCLC tissues and ANCTs	NSCLC tissues *vs.* ANCTs	0.803	82.5	67.5	(31)
	serum of 45 NSCLC patients and 45 healthy controls	NSCLC patients *vs.* healthy controls	0.794	71.1	80.0	
	104 pairs of LUSC tissues	LUSC tissues *vs.* normal tissues	0.774	97.1	51	(44)
	110 pairs of LUSC serum	LUSC serum *vs.* normal tissues	0.789	91.3	60.6	
Oral squamous cell carcinoma (OSCC)	50 OSCC tissues and ANCTs	OSCC tissues *vs.* ANCTs	0.787	68.6	86.0	(33)

ANCTs, adjacent non-cancerous tissues; NSCLC, non-small cell lung cancer; LUSC, lung squamous cell carcinoma.

## Discussion

CircPVT1 is transcribed from a locus that is closely associated with cancer. The lncRNA transcribed from this region has been regarded as a cancer-related transcript (51). Most recently, this circRNA has been acknowledged as an oncogenic transcript. CircPVT1 acts as a sponge for miR-125a, miR-125b, miR-124-3p, miR-30a-5p, miR-205-5p, miR‐423‐5p, miR‐526b, miR-137, miR-145-5p, miR-497, miR-30d/e, miR-455-5p, miR-29a-3p, miR-204-5p, miR-149, miR-106a-5p, miR-377, miR-3666, miR-203, and miR-199a-5p. Moreover, it can regulate activity of PI3K/AKT, Wnt5a/Ror2, E2F2, and HIF-1α. Thus, the sponging role of circPVT1 is the most appreciated function of this circRNA.

The therapeutic potential of circPVT1 has been deduced from altered response of cancer cell lines as well as primary neoplasms to different drugs depending on the expression levels of this circRNA (52). Moreover, independent studies in animal models of gastric cancer, osteosarcoma, lung cancer, medullary thyroid cancer, breast cancer, oral squamous cell carcinoma, hepatocellular carcinoma, and renal cell carcinoma have verified the oncogenic roles of circPVT1. These studies have also shown the effectiveness of circPVT1 silencing in reduction of tumor burden, suggesting novel treatment modalities for further examinations in clinical settings.

Upregulation of circPVT1 has been associated with decreased survival of patients with diverse cancer types, demonstrating the role of this circRNA as a prognostic marker. Two recent meta-analyses have indicated the importance of circPVT1 levels in prediction of malignant behavior of different types of neoplasms (53, 54). Further proofs for participation of circPVT1 in the carcinogenesis have come from the observed association between its levels in tumors and clinical data including TNM stage, tumor size, and lymph node positivity. CircPVT1 has also been suggested as a diagnostic marker in lung cancer as well as oral squamous cell carcinoma. The discovery of presence of circPVT1 in the cancer-derived exosomes not only highlights the biomarker role of this circRNA but also unravels a less-studied route of promotion of malignant behavior in tumor tissues by this circRNA.

Although expression of circPVT1 has been assessed by different methods, based on the poor reproducibility of assessment of circRNA expression levels (55), precise identification and quantification of circPVT1 expression are crucial.

Cumulatively, circPVT1 is implicated in response of cancer patients to chemotherapeutic agents such as cisplatin, doxorubicin, and paclitaxel. Thus, circPVT1 silencing is a putative modality for improvement of chemotherapy response in patients.

## Author Contributions

SG-F wrote the draft and revised it. MT designed and supervised the study. EJ and TK collected the data and designed the figures and tables. All authors contributed to the article and approved the submitted version.

## Conflict of Interest

The authors declare that the research was conducted in the absence of any commercial or financial relationships that could be construed as a potential conflict of interest.

## Publisher’s Note

All claims expressed in this article are solely those of the authors and do not necessarily represent those of their affiliated organizations, or those of the publisher, the editors and the reviewers. Any product that may be evaluated in this article, or claim that may be made by its manufacturer, is not guaranteed or endorsed by the publisher.

## References

[1] Li X Yang L Chen L-L. The Biogenesis, Functions, and Challenges of Circular RNAs. Mol Cell (2018) 71(3):428–42. doi: 10.1016/j.molcel.2018.06.034 30057200

[2] Salzman J Gawad C Wang PL Lacayo N Brown PO. Circular RNAs are the Predominant Transcript Isoform From Hundreds of Human Genes in Diverse Cell Types. PloS One (2012) 7(2):e30733. doi: 10.1371/journal.pone.0030733 22319583 PMC3270023

[3] Vicens Q Westhof E. Biogenesis of Circular RNAs. Cell (2014) 159(1):13–4. doi: 10.1016/j.cell.2014.09.005 25259915

[4] Meng S Zhou H Feng Z Xu Z Tang Y Li P. CircRNA: Functions and Properties of a Novel Potential Biomarker for Cancer. Mol Cancer (2017) 16(1):1–8. doi: 10.1186/s12943-017-0663-2 28535767 PMC5440908

[5] Rajappa A Banerjee S Sharma V Khandelia P. Circular RNAs: Emerging Role in Cancer Diagnostics and Therapeutics. Front Mol Biosci (2020) 7. doi: 10.3389/fmolb.2020.577938 PMC7655967 33195421

[6] Patop IL Wüst S Kadener S. Past, Present, and Future of Circ RNA s. EMBO J (2019) 38(16):e100836. doi: 10.15252/embj.2018100836 31343080 PMC6694216

[7] Pervouchine DD. Circular Exonic RNAs: When RNA Structure Meets Topology. Biochim Biophys Acta (BBA) Gene Regul Mech (2019) 1862(11-12):194384. doi: 10.1016/j.bbagrm.2019.05.002 31102674

[8] Liang D Tatomer DC Luo Z Wu H Yang L Chen L-L. The Output of Protein-Coding Genes Shifts to Circular RNAs When the pre-mRNA Processing Machinery is Limiting. Mol Cell (2017) 68(5):940–54.e3. doi: 10.1016/j.molcel.2017.10.034 29174924 PMC5728686

[9] Ashwal-Fluss R Meyer M Pamudurti NR Ivanov A Bartok O Hanan M. circRNA Biogenesis Competes With pre-mRNA Splicing. Mol Cell (2014) 56(1):55–66. doi: 10.1016/j.molcel.2014.08.019 25242144

[10] Memczak S Jens M Elefsinioti A Torti F Krueger J Rybak A. Circular RNAs are a Large Class of Animal RNAs With Regulatory Potency. Nature (2013) 495(7441):333–8. doi: 10.1038/nature11928 23446348

[11] Yang L Fu J Zhou Y. Circular RNAs and Their Emerging Roles in Immune Regulation. Front Immunol (2018) 9:2977. doi: 10.3389/fimmu.2018.02977 30619334 PMC6305292

[12] Zhang Y Zhang X-O Chen T Xiang J-F Yin Q-F Xing Y-H. Circular Intronic Long Noncoding RNAs. Mol Cell (2013) 51(6):792–806. doi: 10.1016/j.molcel.2013.08.017 24035497

[13] Li Z Huang C Bao C Chen L Lin M Wang X. Exon-Intron Circular RNAs Regulate Transcription in the Nucleus. Nat Struct Mol Biol (2015) 22(3):256–64. doi: 10.1038/nsmb.2959 25664725

[14] Chen J Li Y Zheng Q Bao C He J Chen B. Circular RNA Profile Identifies Circpvt1 as a Proliferative Factor and Prognostic Marker in Gastric Cancer. Cancer Lett (2017) 388:208–19. doi: 10.1016/j.canlet.2016.12.006 27986464

[15] Dudekula DB Panda AC Grammatikakis I De S Abdelmohsen K Gorospe M. CircInteractome: A Web Tool for Exploring Circular RNAs and Their Interacting Proteins and microRNAs. RNA Biol (2016) 13(1):34–42. doi: 10.1080/15476286.2015.1128065 26669964 PMC4829301

[16] Panda AC Grammatikakis I Kim KM De S Martindale JL Munk R. Identification of Senescence-Associated Circular RNAs (SAC-RNAs) Reveals Senescence Suppressor Circpvt1. Nucleic Acids Res (2017) 45(7):4021–35. doi: 10.1093/nar/gkw1201 PMC5397146 27928058

[17] Fang S Zhang L Guo J Niu Y Wu Y Li H. NONCODEV5: A Comprehensive Annotation Database for Long Non-Coding RNAs. Nucleic Acids Res (2018) 46(D1):D308–14. doi: 10.1093/nar/gkx1107 PMC5753287 29140524

[18] Salehi M Sharifi M Bagheri M. Knockdown of Long Noncoding RNA Plasmacytoma Variant Translocation 1 With Antisense Locked Nucleic Acid GapmeRs Exerts Tumor-Suppressive Functions in Human Acute Erythroleukemia Cells Through Downregulation of C-MYC Expression. Cancer Biother Radiopharm (2019) 34(6):371–9. doi: 10.1089/cbr.2018.2510 30141968

[19] Zhang J Chen S Yang J Zhao F. Accurate Quantification of Circular RNAs Identifies Extensive Circular Isoform Switching Events. Nat Commun (2020) 11(1):1–14. doi: 10.1038/s41467-019-13840-9 31900416 PMC6941955

[20] Liu Y-Y Zhang L-Y Du W-Z. Circular RNA Circ-PVT1 Contributes to Paclitaxel Resistance of Gastric Cancer Cells Through the Regulation of ZEB1 Expression by Sponging miR-124-3p. Biosci Rep (2019) 39(12):BSR20193045. doi: 10.1042/BSR20193045 31793989 PMC6928529

[21] Yao W Guo P Mu Q Wang Y. Exosome-Derived Circ-PVT1 Contributes to Cisplatin Resistance by Regulating Autophagy, Invasion, and Apoptosis *via* miR-30a-5p/YAP1 Axis in Gastric Cancer Cells. Cancer Biother Radiopharmaceuticals (2021) 36(4):347–59. doi: 10.1089/cbr.2020.3578 32799541

[22] Wang J Huang K Shi L Zhang Q Zhang S. CircPVT1 Promoted the Progression of Breast Cancer by Regulating MiR-29a-3p-Mediated AGR2-HIF-1α Pathway. Cancer Manage Res (2020) 12:11477. doi: 10.2147/CMAR.S265579 PMC7672658 33223849

[23] Bian Q. Circular RNA PVT1 Promotes the Invasion and Epithelial–Mesenchymal Transition of Breast Cancer Cells Through Serving as a Competing Endogenous RNA for miR-204-5p. OncoTargets Ther (2019) 12:11817. doi: 10.2147/OTT.S180850 PMC7502987 32982273

[24] Sun X Luo L Gao Y. Circular RNA PVT1 Enhances Cell Proliferation But Inhibits Apoptosis Through Sponging microRNA-149 in Epithelial Ovarian Cancer. J Obstet Gynaecol Res (2020) 46(4):625–35. doi: 10.1111/jog.14190 32048451

[25] Liu Y-P Wan J Long F Tian J Zhang C. Circpvt1 Facilitates Invasion and Metastasis by Regulating miR-205-5p/C-FLIP Axis in Osteosarcoma. Cancer Manage Res (2020) 12:1229. doi: 10.2147/CMAR.S231872 PMC7035890 32110097

[26] Wan J Liu Y Long F Tian J Zhang C. Circpvt1 Promotes Osteosarcoma Glycolysis and Metastasis by Sponging miR-423-5p to Activate Wnt5a/Ror2 Signaling. Cancer Sci (2021) 112(5):1707. doi: 10.1111/cas.14787 33369809 PMC8088910

[27] Yan M Gao H Lv Z Liu Y Zhao S Gong W. Circular RNA PVT1 Promotes Metastasis *via* Regulating of miR-526b/FOXC2 Signals in OS Cells. J 5Cell Mol Med (2020) 24(10):5593–604. doi: 10.1111/jcmm.15215 PMC7214167 32249539

[28] Kun-Peng Z Xiao-Long M Chun-Lin Z. Overexpressed Circpvt1, a Potential New Circular RNA Biomarker, Contributes to Doxorubicin and Cisplatin Resistance of Osteosarcoma Cells by Regulating ABCB1. Int J Biol Sci (2018) 14(3):321. doi: 10.7150/ijbs.24360 29559849 PMC5859477

[29] Li D Huang Y Wang G. Circular RNA Circpvt1 Contributes to Doxorubicin (DXR) Resistance of Osteosarcoma Cells by Regulating TRIAP1 *via* miR-137. BioMed Res Int (2021) 2021. doi: 10.1155/2021/7463867 PMC8088374 33981772

[30] Zheng F Xu R. CircPVT1 Contributes to Chemotherapy Resistance of Lung Adenocarcinoma Through miR-145-5p/ABCC1 Axis. Biomed Pharmacother (2020) 124:109828. doi: 10.1016/j.biopha.2020.109828 31986409

[31] Li X Zhang Z Jiang H Li Q Wang R Pan H. Circular RNA Circpvt1 Promotes Proliferation and Invasion Through Sponging miR-125b and Activating E2F2 Signaling in Non-Small Cell Lung Cancer. Cell Physiol Biochem (2018) 51(5):2324–40. doi: 10.1159/000495876 30537738

[32] Qin S Zhao Y Lim G Lin H Zhang X Zhang X. Circular RNA PVT1 Acts as a Competing Endogenous RNA for miR-497 in Promoting Non-Small Cell Lung Cancer Progression. Biomed Pharmacother (2019) 111:244–50. doi: 10.1016/j.biopha.2018.12.007 30590312

[33] He T Li X Xie D Tian L. Overexpressed Circpvt1 in Oral Squamous Cell Carcinoma Promotes Proliferation by Serving as a miRNA Sponge. Mol Med Rep (2019) 20(4):3509–18. doi: 10.3892/mmr.2019.10615 PMC6755181 31485648

[34] Zhu X Du J Gu Z. Circ-PVT1/miR-106a-5p/HK2 Axis Regulates Cell Growth, Metastasis and Glycolytic Metabolism of Oral Squamous Cell Carcinoma. Mol Cell Biochem (2020) 474(1):147–58. doi: 10.1007/s11010-020-03840-5 32737775

[35] Hu J Han Q Gu Y Ma J McGrath M Qiao F. Circular RNA PVT1 Expression and its Roles in Acute Lymphoblastic Leukemia. Epigenomics (2018) 10(6):723–32. doi: 10.2217/epi-2017-0142 29693417

[36] Bu N Dong Z Zhang L Zhu W Wei F Zheng S. CircPVT1 Regulates Cell Proliferation, Apoptosis and Glycolysis in Hepatocellular Carcinoma *via* miR-377/TRIM23 Axis. Cancer Manage Res (2020) 12:12945. doi: 10.2147/CMAR.S280478 PMC7751302 33364841

[37] Li Y Shi H Yuan J Qiao L Dong L Wang Y. Downregulation of Circular RNA Circpvt1 Restricts Cell Growth of Hepatocellular Carcinoma Through Downregulation of Sirtuin 7 *via* microRNA-3666. Clin Exp Pharmacol Physiol (2020) 47(7):1291–300. doi: 10.1111/1440-1681.13273 32017171

[38] Zhu Y Liu Y Xiao B Cai H Liu M Ma L. The Circular RNA PVT1/miR-203/HOXD3 Pathway Promotes the Progression of Human Hepatocellular Carcinoma. Biol Open (2019) 8(9):bio043687. doi: 10.1242/bio.043687 31551242 PMC6777361

[39] Chi G Yang F Xu D Liu W. Silencing Hsa_Circ_PVT1 (Circpvt1) Suppresses the Growth and Metastasis of Glioblastoma Multiforme Cells by Up-Regulation of miR-199a-5p. Artif Cells Nanomed Biotechnol (2020) 48(1):188–96. doi: 10.1080/21691401.2019.1699825 31865777

[40] Zheng Z Chen Z Zhong Q Zhu D Xie Y Shangguan W. CircPVT1 Promotes Progression in Clear Cell Renal Cell Carcinoma by Sponging miR-145-5p and Regulating TBX15 Expression. Cancer Sci (2021) 112(4):1443. doi: 10.1111/cas.14814 33453148 PMC8019224

[41] Chen S Ding J Wang Y Lu T Wang L Gao X. RNA-Seq Profiling of Circular RNAs and the Oncogenic Role of Circpvt1 in Cutaneous Squamous Cell Carcinoma. OncoTargets Ther (2020) 13:6777. doi: 10.2147/OTT.S252233 PMC7367724 32764965

[42] Zhong R Chen Z Mo T Li Z Zhang P. Potential Role of Circpvt1 as a Proliferative Factor and Treatment Target in Esophageal Carcinoma. Cancer Cell Int (2019) 19(1):1–9. doi: 10.1186/s12935-019-0985-9 31636510 PMC6794789

[43] Zhu Y Yang L Chong Q-Y Yan H Zhang W Qian W. Long Noncoding RNA Linc00460 Promotes Breast Cancer Progression by Regulating the miR-489-5p/FGF7/AKT Axis. Cancer Manage Res (2019) 11:5983. doi: 10.2147/CMAR.S207084 PMC6612969 31308741

[44] Shi J Lv X Zeng L Li W Zhong Y Yuan J. CircPVT1 Promotes Proliferation of Lung Squamous Cell Carcinoma by Binding to miR-30d/E. J Exp Clin Cancer Res (2021) 40(1):1–15. doi: 10.1186/s13046-021-01976-w 34112238 PMC8194141

[45] Zheng X Rui S Wang X-F Zou X-H Gong Y-P Li Z-H. Circpvt1 Regulates Medullary Thyroid Cancer Growth and Metastasis by Targeting miR-455-5p to Activate CXCL12/CXCR4 Signaling. J Exp Clin Cancer Res (2021) 40(1):1–17. doi: 10.1186/s13046-021-01964-0 33962657 PMC8106141

[46] Liu W Xiong Y Wan R Shan R Li J Wen W. The Roles of Circmto1 in Cancer. Front Cell Dev Biol (2021) 9. doi: 10.3389/fcell.2021.656258 PMC8277961 34277605

[47] Yuan B Yang J Gu H Ma C. Down-Regulation of LINC00460 Represses Metastasis of Colorectal Cancer *via* WWC2. Digest Dis Sci (2020) 65(2):442–56. doi: 10.1007/s10620-019-05801-5 31541369

[48] Wang Z Su M Xiang B Zhao K Qin B. Circular RNA PVT1 Promotes Metastasis *via* miR-145 Sponging in CRC. Biochem Biophys Res Commun (2019) 512(4):716–22. doi: 10.1016/j.bbrc.2019.03.121 30922567

[49] Kong S Yang Q Tang C Wang T Shen X Ju S. Identification of Hsa_Circ_0001821 as a Novel Diagnostic Biomarker in Gastric Cancer *via* Comprehensive Circular RNA Profiling. Front Genet (2019) 10:878. doi: 10.3389/fgene.2019.00878 31616472 PMC6764484

[50] Lu H Xie X Chen Q Cai S Liu S Bao C. Clinical Significance of Circpvt1 in Patients With Non-Small Cell Lung Cancer Who Received Cisplatin Combined With Gemcitabine Chemotherapy. Tumori J (2021) 107(3):204–8. doi: 10.1177/0300891620941940 32734834

[51] Ghafouri-Fard S Omrani MD Taheri M. Long Noncoding RNA PVT1: A Highly Dysregulated Gene in Malignancy. J Cell Physiol (2020) 235(2):818–35. doi: 10.1002/jcp.29060 31297833

[52] Adhikary J Chakraborty S Dalal S Basu S Dey A Ghosh A. Circular PVT1: An Oncogenic Non-Coding RNA With Emerging Clinical Importance. J Clin Pathology (2019) 72(8):513–9. doi: 10.1136/jclinpath-2019-205891 31154423

[53] Lin Z Tang X Wang L Ling L. Prognostic and Clinicopathological Value of Circpvt1 in Human Cancers: A Meta-Analysis. Cancer Rep (Hoboken NJ) (2021) 1:e1385. doi: 10.1002/cnr2.1385 PMC8551984 33793089

[54] Zhou J Zhang H Zou D Zhou Z Wang W Luo Y. Clinicopathologic and Prognostic Roles of Circular RNA Plasmacytoma Variant Translocation 1 in Various Cancers. Expert Rev Mol Diagnostics (2021) 20:1–10. doi: 10.1080/14737159.2021.1964959 34346262

[55] Ye C-Y Zhang X Chu Q Liu C Yu Y Jiang W. Full-Length Sequence Assembly Reveals Circular RNAs With Diverse Non-GT/AG Splicing Signals in Rice. RNA Biol (2017) 14(8):1055–63. doi: 10.1080/15476286.2016.1245268 PMC5680721 27739910

